# Chloride of Methyl

**Published:** 1891-01

**Authors:** L. E. Custer

**Affiliations:** Dayton, O.


### Chloride of Methyl.

BY L. E. CUSTER, D.D.S., DAYTON, O.

Read before the Ohio State Dental Society, held at Columbus, October, 1890.

Some three years ago Dr. R. Ottolengui suggested the ether spray as an obtundent for sensitive dentine. Although this was not the first time the ether spray was used for this purpose, it was original with him, and by the strong claims for it succeeded in turning the attention of some in that direction which resulted in a number of new agents for obtunding sensitive dentine. Dr. Otto Arnold at a meeting of this society, held two years ago, said he used a spray of nitrous oxide for the purpose, and was followed by Dr. G. L. Curtis, who read a paper upon the same agent at the March meeting of the New York Odontological Society.

The last obtundent of this nature of any importance is chloride of methyl, which was introduced by Dr. M. L. Rhein after a number of efforts.

There has been considerable confusion regarding the action of these agents. Dr. Ottolengui was first led to use the ether spray, by the idea, that it would produce more thorough dehydration of the dentine than could be accomplished by alcohol and hot air, and he was successful. But let me say that this success was not the result of dehydration. 1st, because ether has no affinity for water; 2nd, because it reduces the temperature of the dentine which opposes the evaporation of its moisture. The ether spray unless inhaled obtunds sensitive dentine by no other means than the cold which it produces.

Dr. Curtis in his paper in speaking of the action of nitrous oxide, said: "Whether the action of nitrous oxide, as herein set forth, is chemical or mechanical I am yet unable to state, but by experiments now under progress I hope to arrive at a clear solution of the question. Thus far I am led to the belief that anæsthesia is the result of dehydration." Experiments would be unnecessary if the author would read a lesson from the simple nurse-girl who hangs the napkin before the fire to dry. Heat produces evaporation of moisture, not cold. Chemists say nitrous oxide has no affinity for water, but it has been observed that water takes up 80 per cent. of its volume of the gas. The cold produced by the volatilization of nitrous oxide is so intense that the water of the dentinal tubuli would be frozen before enough could evaporate to produce the profound results following the use of the powerful agent. The only manner in which I see nitrous oxide could be effective in any other way than by reduction of temperature, is either by its being inhaled, or in the following manner: It is said that after the first few inhalations of nitrous oxide the lung membrane becomes anæsthetized, so the 80 per cent. absorbed by the water in the tubule might, in like manner, anæsthetize the fibril. But that is a rather fine point.

There is no use in "chasing the devil around the stump," unless the agent is inhaled in sufficient quantiy to produce general anæthesia, it is cold that does the work, and for the following reason: For the perfect performance of nerve function the normal temperature of the organism is necessary. Lowering of the temperature obtunds the sensibility somethat proportionate to the departure from normality. The distance of the bulbous portion of the pulp from collateral circulation, there being no blood circulation in the dentine, and the ease with which the crown of the tooth may be isolated from surrounding sources of warmth, make the action of refrigerating agents the most effective obtundent of sensitive dentine we have.

Chloride of methyl resembles common ether in appearance, taste, and smell, but it is not so inflammable. It is produced by the action of chlorine upon marsh gas, by heating together common salt, sulphuric acid, and methyl alcohol, or it is more cheaply manufactured by using the waste products of beets used in the manufacture of sugar.

This is an ether having the formula $CH_3Cl$. The hydrocarbon radical is very low in the series and for that reason we find it difficult at ordinary temperature to keep the methyl from passing into the gaseous form. Its boiling point is about 73° Fahrenheit, while that of ether is 96°, rhigoline 64°, and that of nitrous oxide 148° below zero. We therefore have at our command an agent which is between ether and rhigoline, which, by the warmth of the hand, volatilizes with sufficient force to form a continuous spray. It is capable of reducing the temperature to about 40° below zero which gives a range wide enough for all ordinary purposes. It has no affinity for water, and when used as an obtundent for sensitive dentine it acts purely by extracting the heat.

This medicament has another property I have found, but of which I have seen no mention, and that is as a general anæsthetic. I have had no opportunity of experimenting upon any lower animal than myself, but so far as I can ascertain it is quite as prompt and powerful as either chloroform or ether. I did not test its full capacity or I would probably not have been here.

There is one thing in its favor as a general anæsthetic, however, and that is this: The theory of Dr. H. C. Wood is, that chloroform causes more deaths than ether because it is not as volatile an agent; that when it is removed at the outset of alarming symptoms, that which is already in the system continues to act for some time, and occasionally till syncope results, while ether being more volatile, when not inhaled, readily passes off. Now, chloride of methyl being still more volatile than either of these, might it not be found a safer anæsthetic?

Dr. M. L. Rhein, to whom belongs the credit of introducing it to the dental profession, uses this agent for facial neuralgia and sensitive dentine, and it may also be used for freezing histological specimens, but I will give its qualities as an obtundent of sensitive dentine.

Volatilizing at ordinary temperature it requires no apparatus for generating a blast as with ether. Since it is not a solvent of caoutchouc, it may be carried to the tooth through a rubber tube and its flow regulated by a thumb-screw. It produces more intense cold than ether, and on that account the pain following its application is more brief, and the obtunding effect of much longer duration. It, therefore, also requires less time to obtain the same results secured by ether. It volatilizes more rapidly than ether so that all the adjoining parts are not smeared with the agent.

The objections to chloride of methyl as an obtundent to sensitive dentine are, that it is a general anæsthetic and very likely to be inhaled during the refrigerating process. It is also at the present time rather expensive. Altogether I think it is all that Dr. Rhein has claimed for it as an obtundent, and is to be preferred as a cold producing agent for sensitive dentine; but the older we get, and the more experience we have, the better, I think, we can control our patients by a sort of personal magnetism, and the use of dehydrating agents, such as alcohol and hot air, will in nearly all cases be sufficient without resorting to such dangerous methods as the use of refrigerants.

